# Chronic Pulmonary Aspergillosis Secondary Infection: A Case Report of *Achromobacter* spp. Lung Infection

**DOI:** 10.1002/ccr3.71121

**Published:** 2025-10-06

**Authors:** Marius Paulin Ngouanom Kuate, Felix Bongomin, David W. Denning

**Affiliations:** ^1^ Department of Microbiology and Parasitology, Faculty of Science University of Buea Buea Cameroon; ^2^ Department of Medical Microbiology and Immunology, Faculty of Medicine Gulu University Gulu Uganda; ^3^ Manchester Fungal Infection Group, Faculty of Biology, School of Biological Sciences, Medicine and Health The University of Manchester Manchester UK; ^4^ The Global Action for Fungal Infections Genève Switzerland

**Keywords:** *Achromobacter* spp., *Aspergillus*‐specific IgG/IgM antibody, Cameroon, COVID‐19, CPA, PTB

## Abstract

Both pulmonary tuberculosis (PTB) and coronavirus disease‐2019 (COVID‐19) are risk factors for chronic pulmonary aspergillosis (CPA) and other pulmonary infectious diseases because of residual lung damage. We report a case of *Achromobacter* spp. infection following CPA in an immunocompetent woman with a history of PTB and COVID‐19. A 63‐year‐old Cameroonian woman presented in November 2021 with a history of cough with productive muco‐purulent sputum, asthenia, headaches, and chest pain for 8 weeks. There was no history of hemoptysis or difficulty in breathing. She was treated for PTB in 2002 and COVID‐19 in 2020 and had no other underlying co‐morbidities. Chest X‐ray showed bronchiectasis in the right lung and features of healed PTB. SARS‐CoV‐2 antigen, antibody, and real‐time polymerase chain reaction tests were negative. Microscopy and GeneXpert MTB/RIF on the sputum sample were both negative. Sputum samples grew *Aspergillus flavus* complex and *Aspergillus niger* complex, and serum *Aspergillus*‐specific IgG‐IgM antibody was positive, suggestive of CPA. She showed significant clinical improvement on itraconazole tablets 200 mg (every 12 h) after 4 months of therapy. She presented 1 month later with severe symptomatic relapse and elevated white blood cells (27,000 cells/μL). Antibiotic therapy with amoxicillin + clavulanic acid and subsequently with ceftriaxone was unsuccessful. Chest CT scan showed a middle right mediastinal tissue mass with crenulated edges. Bronchoalveolar lavage (BAL) and lung biopsy testing yielded a negative result for PTB, invasive aspergillosis, and lung cancer. However, the BAL sample grew *Achromobacter* spp. She was initiated on imipenem 1 g (3 g/day × 10 days) with resolution of symptoms. This case suggests that because of the high burden of TB and COVID‐19 in Cameroon, pulmonary bacterial and fungal superinfections are underreported. CPA is presently undiagnosed and underreported in Cameroon. Further investigations should be performed in patients not responding to usual antibiotics.

**Summary**

- CPA can mimic and is often misdiagnosed as PTB in endemic areas.
- Bacterial superinfection may occur, sometimes with unusual pathogens such as *Achromobacter* spp.

## 1 Introduction

Aspergillosis is a spectrum of disorders caused by the ubiquitous, opportunistic pathogen of the genus *Aspergillus*, which affects both humans and animals [1]. Infection commonly results from inhalation of conidia present in the environment [2]. Chronic pulmonary aspergillosis (CPA) is often seen in predominantly immunocompetent individuals with residual lung diseases, such as treated pulmonary tuberculosis (PTB) [3].

TB is highly prevalent in Africa, with an estimated prevalence of about 220 cases per 100,000 people [4]. In Cameroon, the incidence was estimated to be about 174 cases per 100,000 population in the 2020 WHO Global TB Report [5]. Previously treated PTB is an important risk factor for pulmonary fungal infections [6]. The global prevalence of CPA secondary to TB is estimated to lie between 0.8 and 1.37 million cases [7].

On the other hand, coronavirus disease −2019 (COVID‐19) probably predisposes to CPA and other pulmonary fungal diseases because of residual lung damage. Post‐COVID‐19 aspergillosis has been reported in small case reports and series from across the world [8, 9, 10, 11], but none in Cameroon. Most COVID‐19‐associated fungal infections are reported to occur 2 weeks after the appearance of the first symptoms of COVID‐19 [9]. Moreover, aspergillosis following pulmonary tuberculosis is considered a significant public health problem and widely reported [12, 13, 14, 15], but what is the situation of post‐CPA bacterial infections? One of the rare studies addressing this issue is a retrospective study, which found bacteria in 73 (51.4%) patients, 11 (7.7%) of whom had multiple microbial species [10]. Here, we report a case of *Achromobacter* spp. infection following CPA in a woman with a history of PTB and COVID‐19.

## 2 Case History/Examination

A 63‐year‐old Cameroonian woman presented to the chest clinic in November 2021 with a 2‐month history of productive cough, asthenia, headache, and chest pain. Her medical history was significant for severe COVID‐19 diagnosed in July 2020 and PTB in 2002 in the background of controlled systemic arterial hypertension. Her weight was 105 kg and blood pressure 140/83 mmHg.

**FIGURE 1:**
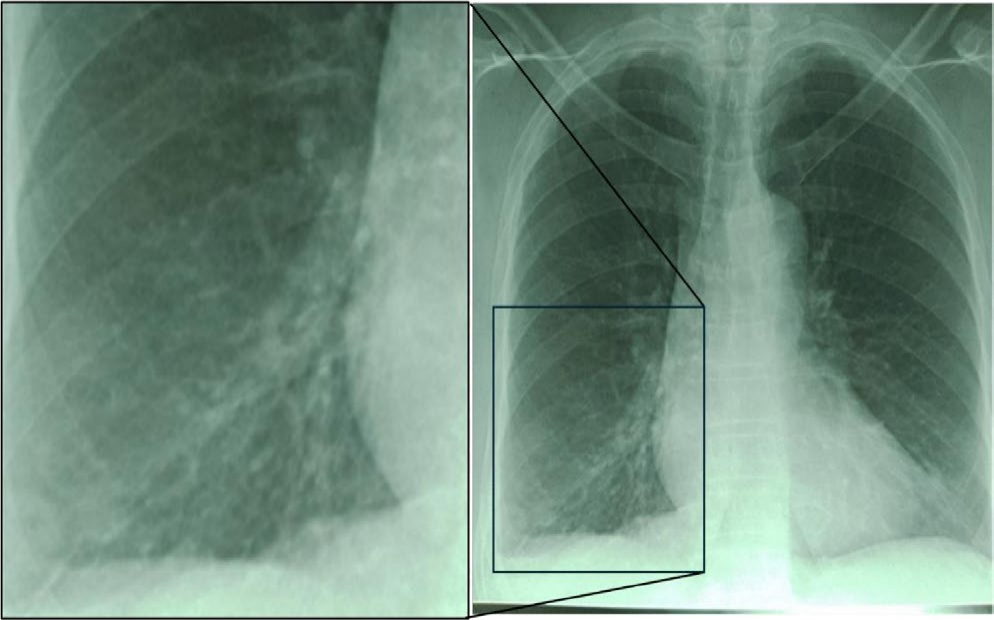
Patient's chest X‐ray showing bronchiectasis with a small infiltrate (probable consolidation) at the left base just adjacent to the L heart border.

On laboratory investigations, SARS‐CoV‐2 antigen and antibodies and real‐time polymerase chain reaction (RT‐PCR) tests were all negative. Two sputum samples stained with auramine‐rhodamine did not show any *Mycobacterium tuberculosis* . Her chest X‐ray showed bronchiectasis in the right lung, mostly proximal in the upper lobe and linearly from the hilum to the costophrenic angle. There was a small cavity at the right costophrenic angle, some less distinct densities on the left lower lobe, and just above the left hilum, some features of old TB, possibly bronchiectasis (Figure 1). Her electrocardiogram was normal. Her blood biochemistry showed sodium of 134 mEq/L (normal range (NR): 135–148), calcium 80 mg/L (NR: 85–105), magnesium 15 mg/L (NR: 16–25), creatinine 13 mg/L (NR: 6–11), and urea 0.25 g/L (NR: 0.10–0.4). Random blood sugar was 1.0 g/L (NR: 0.1–1.1). The full blood count was also normal. She was initially treated as a case of bacterial pneumonia with antibiotics of different spectrum and mechanism of action (Table 1; amoxicillin 875 mg + clavulanic acid 125 mg, 1 g intravenous every 8 h, azithromycin 500 mg, one tablet each every 24 h and gentamicin 160 mg one vial intramuscular every 24 h) from day 3 to day 18 with no improvement.

**TABLE 1:** Mode and spectrum of action of drugs used during the case management.

Drugs	Spectrum of action	Mode of action	Comments	References
Amoxicillin Clavulanate	Gram‐positive and Gram‐negative bacteria, beta‐lactamase‐producing Gram‐positive and Gram‐negative organisms, including some Gram‐negative anaerobes	Clavulanate prevents bacterial degradation of β‐lactams by deactivating β‐lactamases, ensuring amoxicillin's antimicrobial mechanism remains intact	Infectious Diseases Society of America (IDSA) recommends this combination medication over amoxicillin alone	[16]
Azithromycin	Acute bacterial sinusitis, community‐acquired pneumonia and pharyngitis/tonsillitis	Second generation macrolide antibiotic which inhibits bacterial protein synthesis, quorum‐sensing and reduces the formation of biofilm	It is well‐tolerated and has a very good record of safety	[17, 18]
Gentamicin	An aminoglycoside antibiotic active against aerobic gram‐negative such as Enterobacteriaceae family, *Pseudomonas aeruginosa*, and some strains of *Neisseria*, *Moraxella*, and *Haemophilus* genera	It is bactericidal and passes through the gram‐negative membrane in an o*xygen‐dependent* active transport	Prescription of gentamicin maybe appropriate when based on epidemiological data.	[19, 20]
Itraconazole	Itraconazole has efficacy against a variety of fungal infections, including blastomycosis, histoplasmosis, aspergillosis, sporotrichosis, candidiasis, and onychomycosis	Itraconazole exhibits fungistatic (slows the growth) activity against yeast‐like fungi and fungicidal (kills the fungus) activity against some strains of *Aspergillus* spp	FDA has approved it for the treatment blastomycosis, histoplasmosis, and aspergillosis	[21, 22]
Imipenem	Has a wide spectrum of antibacterial activity against gram‐negative and gram‐positive aerobic and anaerobic bacteria, including many multiresistant strains.	It has a bactericidal effect by inhibiting cell wall synthesis.	Imipenem is commonly used in combination with cilastatin and is now available in a triple‐drug product with cilastatin and relebactam	[23, 24, 25]

## 3 Differential Diagnosis, Investigations, and Treatment

After the course of antibiotics, she developed a cough productive of mucopurulent sputum. We had a working diagnosis of a possible SARS‐COV‐2 re‐infection and PTB, with a differential diagnosis of bacterial pneumonia, CPA, nocardiosis, malignancies, and others.

**FIGURE 2:**
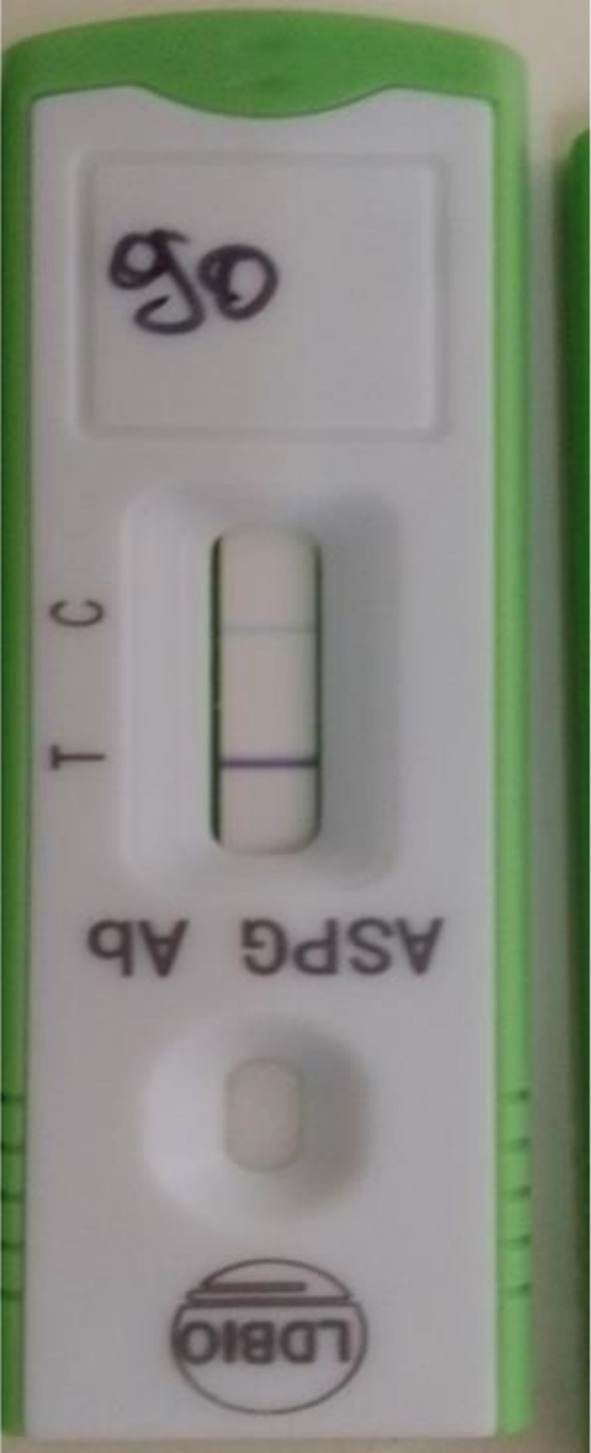
Patient's serum positive for *Aspergillus*‐specific IgG/IgM LFA, indicative of a probable CPA.

A second COVID‐19 antigen and RT‐PCR tests came out negative. Her sputum sent for culture grew *Aspergillus flavus* and *Aspergillus niger*. This was followed by an *Aspergillus*‐specific IgG/IgM antibody lateral flow assay (LFA) testing on serum, which was also positive (Figure 2), suggestive of CPA. She was initiated on itraconazole tablets 200 mg (one tablet every 12 h) and advised on an appropriate diet to enhance itraconazole absorption. She showed significant clinical improvement after 4 months of therapy. She continues long‐term care and monitoring of itraconazole adverse events in the clinic. However, she presented just 1 month later with severe symptoms relapse and elevated white blood cells (27,000 cells/μL). A 15 days therapy with intravenous amoxicillin (875 mg) + clavulanic acid (125 mg) and subsequently intravenous ceftriaxone (1 g) was unsuccessful. Chest CT‐scan showed a middle right mediastinal tissue mass with crenulated edges. Testing of bronchoalveolar lavage (BAL) and lung biopsy, both obtained through bronchoscopy, yielded a negative result for PTB, invasive aspergillosis, and lung cancer. However, BAL grew *Achromobacter* spp., which was resistant to the majority of the antibiotics tested and intermediate to imipenem, ceftazidime, and piperacillin (Table 2). She was initiated on imipenem 1 g (three times a day for 10 days) with a resolution of symptoms and a negative chest CT‐scan 3 weeks after treatment.

**TABLE 2:** Result of susceptibility testing of antibiotics against *Achromobacter* spp.

Antibiotic tested	Susceptibility result
Aztreonam	Resistant
Ticarcillin	Resistant
Piperacillin	Intermediate
Ceftazidime	Intermediate
Imipenem	Intermediate
Tobramycin	Resistant
Amikacin	Resistant
Ciprofloxacin	Resistant

## 4 Discussion

In this report, we describe a case of *Achromobacter* spp. infection following CPA in a woman with a history of PTB and COVID‐19. *Achromobcter* spp. are naturally distributed environmental pathogens emerging as human pathogens. They are associated with several types of infections, including pneumonia [26, 27, 28, 29]. They are resistant to a variety of antibiotics, including aminoglycosides, some monobactams, tetracyclines, some penicillins (penicillin G, ticarcillin), and cephalosporins. Their sensitivity to antibiotics is limited to trimethoprim‐sulfamethoxazole, ceftazidime, piperacillin, and carbapenems, with imipenem the most active [26, 28, 30]. To our knowledge, no such association of CPA with *Achromobacter* spp. infection has been previously described. CPA is a long‐term *Aspergillus* infection of the lung, usually but not exclusively caused by *Aspergillus fumigatus* . Unlike invasive forms of aspergillosis, CPA affects immunocompetent people with pre‐existing or co‐existing lung disease such as TB [31]. In Cameroon, a 2018 prevalence estimate of CPA modeled on cases following PTB was 4983 cases (22/100,000) [32]. Diagnosis of CPA in our patient was performed following the guidelines proposed by Denning et al., which defined CPA as illness for > 3 months and all of the following: (1) weight loss, persistent cough, and/or hemoptysis; (2) chest images showing progressive cavitary infiltrates and/or a fungal ball and/or pericavitary fibrosis or infiltrates or pleural thickening; and (3) a positive *Aspergillus* IgG assay result or other evidence of *Aspergillus* infection [33]. Our patient had an illness for more than 3 months, a persistent cough, a remarkable chest radiography, a negative TB by smear, and a positive *Aspergillus* IgG assay and culture.

Patients with CPA who deteriorate despite antifungal therapy have a high chance of being infected with bacteria as a co‐infection, especially if they have concurrent bronchiectasis. Also, COVID‐19 is reported to be a risk factor for bacterial infections [34], which may occur months after the COVID‐19 infection [35]. In this report, our patient had the *Achromobacter* infection almost 2 years after COVID‐19.

*Achromobacter* isolated here following CPA was resistant to common antibiotics and intermediate to imipenem, ceftazidime, and piperacillin. An explanation for this may be the natural development of resistance due to the frequent use of common antibiotics in agriculture [36, 37, 38]. The highest dosage of imipenem (3 g/day) [25] was administered to avoid any possible development of resistance in an already intermediate susceptible strain.

## 5 Conclusions

CPA is presently underdiagnosed and underreported in Cameroon. Residual lung damage due to COVID‐19 or PTB, joined to other chronic pulmonary infections such as CPA, predisposes patients to superinfections. Further investigations should be performed in patients not responding to usual antibiotics.

## Author Contributions

**Marius Paulin Ngouanom Kuate:** conceptualization, data curation, funding acquisition, investigation, project administration, writing – original draft, writing – review and editing. **Felix Bongomin:** writing – original draft, writing – review and editing. **David W. Denning:** conceptualization, project administration, writing – review and editing.

## Consent

Written informed consent was obtained from the patient to publish this report in accordance with the journal's patient consent policy.

## Conflicts of Interest

The authors declare no conflicts of interest.

## Data Availability

The authors have nothing to report.
